# The total polyphenolic glycoside extract of *Lamiophlomis rotata* ameliorates hepatic fibrosis through apoptosis by TGF-β/Smad signaling pathway

**DOI:** 10.1186/s13020-023-00723-x

**Published:** 2023-02-24

**Authors:** Guoguo Wan, Zhiwei Chen, Lei Lei, Xiaoyu Geng, Yi Zhang, Congwen Yang, Wenfu Cao, Zheng Pan

**Affiliations:** 1 Chongqing Key Laboratory of Traditional Chinese Medicine for Prevention and Cure of Metabolic Diseases, College of Traditional Chinese Medicine, Chongqing Medical University, No.1, Yixueyuan Road, Yuzhong District, Chongqing, 400016 People’s Republic of China; 2 Centre for Academic Inheritance and Innovation of Ethnic Medicine, Chengdu University of Traditional Chinese Medicine, Chengdu, 611130 China

**Keywords:** Polyphenolic glycosides, *Lamiophlomis rotata*, Hepatic fibrosis, Apoptosis, TGF-β/Smad

## Abstract

**Background:**

Hepatic fibrosis is characterized by the excessive deposition of extracellular matrix (ECM) which is mainly secreted by activated hepatic stellate cells (HSCs). *Lamiophlomis rotata* (*L. rotata*) was recorded to treat jaundice in the traditional Tibetan medical system with the potential of hepatoprotection. However, the bioactivities and the possible mechanism of *L. rotata* on hepatic fibrosis is still largely unknown.

**Aim of the study:**

To investigate the anti-hepatic fibrosis effects of bioactivities in *L. rotata* and the probable mechanism of action.

**Materials and methods:**

Herein, total polyphenolic glycosides of *L. rotata* (TPLR) was purified with the selectivity adsorption resin and was analyzed by ultrahigh-performance liquid chromatography coupled with time-of-flight mass spectrometry (UPLC-Q/TOF/MS^n^). The anti-hepatic fibrosis effect of TPLR was evaluated by carbon tetrachloride (CCl_4_)-induced liver fibrosis, and was evaluated with the apoptosis of activated HSCs.

**Results:**

In total, sixteen compounds, including nine phenylpropanoids and six flavonoids, were identified in the UPLC-TOF-MS^n^ profile of the extracts. TPLR significantly ameliorated hepatic fibrosis in CCl_4_-induced mice and inhibited HSCs proliferation, Moreover, TPLR notably increased the apoptosis of activated HSCs along with up-regulated caspase-3, -8, -9, and -10. Furthermore, TPLR inhibited TGF-β/Smad pathway ameliorating hepatic fibrosis though downregulation the expression of Smad2/3, Smad4, and upregulation the expression of Smad7 in vivo and in vitro. Simultaneously, the expression of fibronectin (FN), α-smooth muscle actin (α-SMA), and Collagen I (Col1α1) were decreased in tissues and in cells with TPLR administration.

**Conclusion:**

These results initially demonstrated that TPLR has the potential to ameliorate hepatic fibrosis through an apoptosis mechanism via TGF-β/Smad signaling pathway.

**Graphical Abstract:**

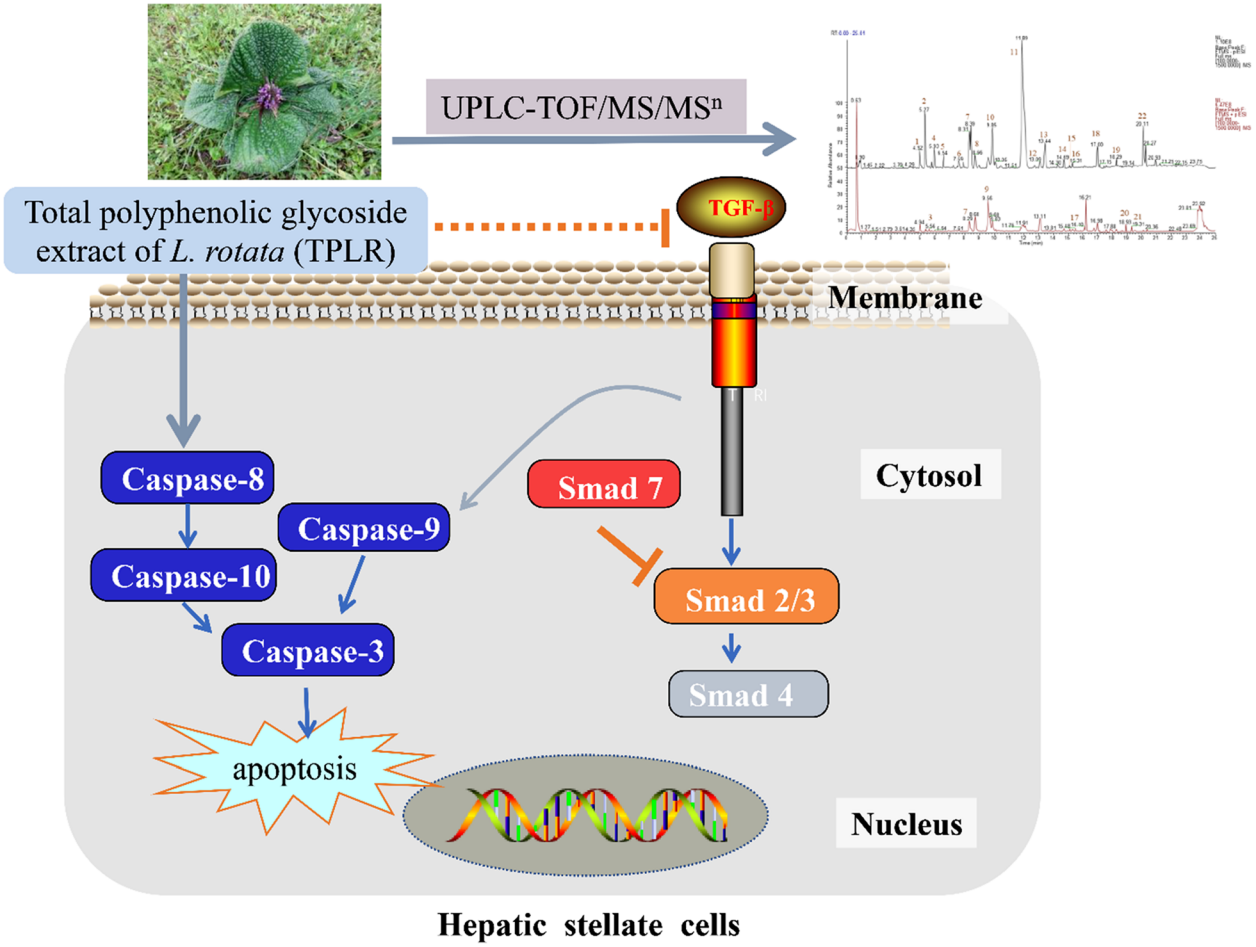

**Supplementary Information:**

The online version contains supplementary material available at 10.1186/s13020-023-00723-x.

## Highlights

- Chemical composition of TPRL was identified by UPLC-Q/TOF/MS^n^ in the profile of total ion current.
- TPRL was initially demostrated the effects of anti-hepatic fibrosis *in vitro* and *in vivo*.
- TPRL ameliorates hepatic fibrosis through apoptosis by TGF-β/Smad signaling pathway.

**Supplementary Information:**

The online version contains supplementary material available at 10.1186/s13020-023-00723-x.

## Introduction

Liver fibrosis is an abnormal hyperplasia of liver connective tissue defined as excess deposition of ECM in response to various liver damages and ultimately progresses to decompensated cirrhosis or hepatocellular carcinoma (HCC) with limited therapeutic options [1]. Activated HSCs are the key effectors of fibrogenesis through increased deposition of fibrillar ECM which negatively affects the regeneration of the liver parenchyma [2, 3]. In the recovering liver, apoptosis is orchestrated by complex interactions between pro-apoptotic and pro-survival signals in activated HSCs, where about 50% of activated HSCs undergo apoptosis [4]. Apoptosis of activated HSCs is a mechanism of cellular recycling and homeostasis that contributes to the spontaneous resolution of liver fibrosis [5, 6]. Nowadays, studies have been conducted to elucidate the molecular mechanisms and the central elements of HSC apoptotic pathways, and different drugs focused on investigations for the activation of apoptosis on HSCs and that needs further studies [7–9].

Recent literature has been conducted to elucidate the molecular mechanisms of natural products on activation and apoptosis of HSCs [10–12]. Aside from caspase, Fas, and TNF-related apoptosis-inducing ligand (TRAIL) pathways, the transforming growth factor-β (TGF-β) signaling is also responsible for regulating HSC apoptosis in the hepatic fibrosis model [13]. TGF-β1-activated HSCs are resistant to apoptosis, and apoptosis resistance aids in maintaining the profibrotic environment and sustains the presence of activated HSCs [14, 15]. Conversely, inhibition of the TGF-β1 signaling pathway substantially promotes the apoptosis of HSCs and ameliorates hepatic fibrosis. For example, Smad7, a negative regulator of Smad signaling involving TGF-β, disrupted cell apoptosis by blocking the binding of TGF-β receptors of Smad2/3 [16]. Additionally, TGF-β1 is one of the most effective cytokines for the transformation and proliferation of HSCs with the upregulation of α-SMA, COL1α1 and FN [17]. Therefore, it is prospective to find compounds or extracts which promote the apoptosis of HSCs through inhibition TGF-β/Smad signaling pathway against hepatic fibrosis in traditional Chinese medicine.

*Lamiophlomis rotata* (*L. rotata*), an orally available Tibetan herb, is known as “Daba” or “Dabuba” in *Jingzhubencao* (Qing Dynasty, AD 1848) and as “Duyiwei” in the Chinese Pharmacopoeia [18]. According to traditional Tibetan medical system, the herb was used to treat “yellow-water disease” such as skin disease, rheumatism, and jaundice with low toxicity for thousands of years [19]. Pharmacological studies found that *L. rotata* has a wide range of activities including analgesic, anti-nociceptive, haemostatic, and anti-inflammatory effects about neuropathic pain, rheumatoid arthritis, and traumatic injury symptoms, while few of these have been concerned to the active ingredients with hepatoprotective effects about jaundice [18, 19]. Fortunately, the aqueous extract of the herb showed hepatoprotective effects with efficiently reduced the levels of AST, ALT in rats [20]. Furthermore, the aqueous extract of *L. rotata* has been demonstrated against hepatic fibrosis in vivo [21]. However, it is still poor knowledge about which kind of extracts or compounds in *L. rotata* are responsible for the effects of anti-hepatic fibrosis, and the probable mechanism of the action still remains largely unknown.

Phytochemical studies on *L. rotata* have identified 223 chemical constituents including phenylpropanoids and flavonoid glycosides (also known as polyphenolic glycoside), iridoids, and volatile oils, in which iridoids and polyphenolic glycosides are considered the main characteristic constituents of *L. rotata* [19]. Our previous study confirmed that iridoid glycosides of *L. rotata* accelerated the secretion of ECM in human dermal fibroblast during the progression of wound healing [22]. Moreover, polyphenolic glycoside extracts have been reported with hepatoprotective effects in *Cistanche deserticola* [23], *Forsythia suspensa* [24], *and Lespedeza cuneate* [25], these results implied that polyphenolic glycosides (including phenylpropanoids and flavonoid glycosides) of *L. rotata* might exert antifibrotic effects. Here, the polyphenolic glycosides extract of *L. rotata* (TPLR) was purified with the selectivity adsorption resin, and was analyzed by UPLC-Q/TOF/MS^n^, experiments were designed to investigate whether TPLR could ameliorate hepatic fibrosis and promote the apoptosis of HSCs by inhibiting the TGF-β/Smad signaling pathway in vivo and in vitro. The study would be beneficial to exploration bioactivities of *L. rotata* against hepatic fibrosis through TGF-β/Smad signaling pathway in traditional Tibetan medicine.

## Materials and methods

### Chemical and regents 

Serum aspartate aminotransferase (AST), the alanine aminotransferase (ALT) and hydroxyproline (Hyp) were manufactured by Jiancheng Bioengineering Institute (Nanjing, China). Anti-αSMA, anti-COL1α1, anti-FN and anti-TGF-β1were brought from Servicebio (Wuhan, China). CCK-8 kit was purchased from Dojindo Laboratories (Kumamoto, Japan). Penicillin and streptomycin, Dulbecco’s modified Eagle’s medium (DMEM) media and FBS were brought form Gibco (Carlsbad, USA). The Annexin V/PI Cell Apoptosis Detection Kit was brought from BD (Pharmingen, USA). RIPA lysis buffer (p0013B), phenylmethanesulfonyl fluoride (PMSF), BCA protein assay kit, SDS-PAGE and EdU Cell Proliferation Kit were got from Beyotime (Beijing, China). 0.25 μm polyvinylidene fluoride membranes (PVDF) were brought from Millipore (Bedford, Massachusetts). Skim milk was provided by Sangon Biotech (Shanghai, China). Primary antibodies against GAPDH, Smad2/3, Smad2, caspase-8, -9, -3 were from Cell Signaling Technology (Boston, USA), primary antibodies against β-actin and Smad7 were purchased from Proteintech (Wuhan, China). Primary antibody against caspase-10 was brought from GeneTex SAN Antonio (Texas, USA). TGF-β1 was purchased from PeproTech (Suzhuo, China) and used at a concentration of 10 ng/ml. TRIzol Reagent (Takara, JAPANES), MonScript™ RTIIIAll-in-One Mix with dsDNase (Monad Biotech, Wuhan, China), and GoTaq^®^ qPCR Master Mix (Promega, USA) were used in RT-qPCR. All other chemicals were commercially available and used without further purification.

### Plant materials and sample preparation

The aerial parts of *L. rotata* (2.4 kg) were collected in Gansu provinces of China. The herbs were authenticated by Professor Yi Zhang (Chengdu University of Traditional Chinese Medicine, Chengdu, China), and were dried in the shade, voucher specimens were deposited at the College of Ethnic Medicine (Chengdu University of Traditional Chinese Medicine, Chengdu, China). The sample was extracted and purified as reported elsewhere [26]. Briefly, the sample was extracted three times with refluxing 70% EtOH, the solvent was removed under reduced pressure, the residue solution was adsorbed and purified by polyamide resin, and the 85% ethanolic elution was removed under reduced pressure and the resulting extract (101.8 g) was the total polyphenolic glycosides extract of *L. rotata.* Samples of suitable concentration (0.1 g/mL) were stored at -4 °C for further analysis. The sample solutions were filtered through a 0.22 **-**µm pore size nylon membrane filter before injection into the UPLC.

### UPLC-MS / MS^n^ analysis

UPLC-MS/MS^n^ analysis was performed according to the method described in our previous studies [27]. The difference between the referential method was the mobile phases system, in this study, the mobile phases were (a) water with 0.1% (v/v) formic acid and (b) methanol with 0.1% (v/v) formic acid, and the optimized elution conditions were as follows: holding at 15% B for 2 min, a linear gradient from 20 to 72% B (2 to 30 min), and then back to15% B in 1 min. The flow rate was 0.3 mL/min. The column temperature was 35 ℃, and the injection volume was 2 µL.

### Animals and treatment 

Fifty mice were randomly distributed into five groups (n = 10), mice in control group were injected with olive oil, other mice were injected with 10% CCl_4_ in olive oil (0.5 ml/kg) twice a week for 8 weeks to induce liver fibrosis [28]. According to the yield of the TPLR preparation and the usual clinical doses for the granule preparation of *L. rotata* [21], the dosages of TPLR in different groups were 50, 100 and 200 mg/kg (equal to 1.0, 2.0 and 4.0 g /kg of raw material). Fourth week after the modeling, each group were orally administered with or without TPLR dissolved in 0.5% sodium carboxymethyl-cellulose (CMC-Na) for 6 weeks.

### Serum aminotransferase and hepatic hydroxyproline measurements 

The levels of ALT, AST, and Hyp in the serum were measured by the instructions of manufacturer (Nanjing Jiancheng Bioengineering Research Institute, Nanjing, China).

### Morphology and immunofluorescence staining

The harvested liver tissues were soaked in 4% polyformaldehyde dehydrated with an ethanol gradient, embedded in paraffin, and the paraffin tissue Sects. (5 µm) were stained with hematoxylin and eosin (HE), Masson, Sirius red and immunofluorescence (IF). Liver morphology and IF were performed as reported elsewhere [29]. Briefly, the liver sections were dewaxed, rehydrated, and placed in 1× citrate buffer (Thermo Fisher Scientific, Shanghai, China) for high pressure antigen repair over 5 min. The sections were then allowed to cool naturally. After the liver tissues were blocked with 5% BSA for 1 h, then incubated with TGF-β1 (1:200), α-SMA (1:500), COL1α1 (1:500), FN (1:200) at 4 °C overnight. After three washes with PBS, the corresponding secondary antibodies (Life Technologies, USA) were applied at a dilution of 1:200 in 1% BSA and incubated with the sections for 1 h at room temperature. Sections were subsequently counter-stained with DAPI for 5 min to visualize nuclei. Images were captured using a Nikon confocal microscope (Nikon, Tokyo, Japan). Three to five fields were randomly selected from each slide, and the proportions of the positive areas or cells in each field were determined using Image J (Media Cybernetics, Silver Springs, MD, USA).

### Cell culture and treatment 

The human hepatic stellate cell line LX-2 and the rat hepatic stellate HSC-T6 cell line were purchased from Fu Heng Cell Center (Shanghai, China and used for in vitro experimental validation. L929 cells, the fibroblast of mice, were brought from Jennio Biological Technology company (Shanghai, China) and applied for cytotoxicity assessments. They were propagated in DMEMmedia supplemented with 10% FBS, 1% penicillin and streptomycin at 37 °C in a humidified atmosphere of 95% air and 5% CO_2_, and all of them were cultured in 10 cm dishes, all cells were cultured until 80% confluent and then used in further applications.

### Cell viability assay

The cell viability and cytotoxicity assessments of different concentrations 0, 10, 25, 50, 100 and 200 μg/mL of TPLR dissolved in 0.2% DMSO was evaluated using the CCK-8 assay, as reported elsewhere [30]. Briefly, the cells were assayed in 96‐well plates at 5000 cells per well by CCK-8 kits and treated with TPLR for 48 h in medium containing 10% serum. After incubation of the cells, viable cells were stained with CCK-8 (10 µl, 3.5 h). Absorbance was measured at 450 nm using a multimode microplate reader (Tecan, Research Triangle Park, USA).

### Edu staining 

Cell proliferation was detected by EdU Cell Proliferation Kit. LX-2 cells with 0, 50, 100 and 200 μg/ml TPLR, respectively, for 48 h. Then, 10 µmol/L EdU were added into cells for 2.5 h followed by washing with pre-cooled PBS. After incubating, LX-2 cells were fixed and permeabilized with 0.1% Triton. Click Additive Solution was incubated for 30 min at room temperature shielded from light [31]. The images were taken by fluorescence microscopy under an optical microscope at 200 × .

### Flow cytometry analysis 

LX-2 cells were treated with consecutive concentrations of TPLR for 48 h. Besides, LX-2 cells were treated with or without 10 ng/mL TGF-β1 12 h, and then incubated with 0, 100 and 200 μg/mL TPLR for 12 h [33]. After incubated for the indicated time, the cells were digested by trypsin without ethylene diamine tetraacetic acid (EDTA), washed with PBS and stained with the Annexin V/PI Cell Apoptosis Detection Kit (BD, Pharmingen, USA), as reported elsewhere [32]. Fluorescence-activated cell sorting (FACS) analysis was acquired from cytometry (BD, California, USA) to analyze the apoptotic cells.

### Real-Time quantitative PCR

The concentration of RNA was determined by absorption measurements at 260/280 nm using a UV–visible spectrophotometer (Bio-Rad, USA). Total RNA was extracted from LX-2/HSC-T6 cells and liver tissue using TRIzol Reagent, and the cDNA was synthesized using MonScript™ RTIIIAll-in-One Mix with dsDNase, from 0.3 μg RNA for each sample. Real-time PCR was performed using a GoTaq® qPCR Master Mix as follows: 10 μL reaction solution contained 5 μL SYBR Mix, 0.2 μL sense and 0.2 μL antisense primers solution (from 10 μM), 1 μL diluted cDNA, and 3.2 μL nuclease-free water. Primer sequences of all selected genes are shown in the Additional file 1: Table S1. The results were quantified using the 2^−ΔΔCt^ method.

### Western blot assay

Liver tissues and LX-2 cells were washed three times with ice-cold PBS and lysed in 100 to 300 μl RIPA lysis buffer (p0013B) supplemented with 1% PMSF [32]. The supernatants of cell lysates were collected by centrifugation at 12,000 × *g* for 10 min at 4 °C, and total protein concentration was measured using the BCA protein assay kit. The extracted proteins were separated by polyacrylamide 8%-12% SDS-PAGE (concentrated gel voltage was 80 V, separated gel voltage was 120 V); and electrophoretically transferred onto 0.25 μm PVDF; use 5% skim milk to block the PVDF for 1 h at room temperature. After washing the membrane with TBST, incubate with the following antibodies at 4 °C in a shaker overnight, GAPDH, Caspase-3, -8, -9, -10, Smad2/3, and Smad4 (1: 1000), β-actin (1: 10,000), Smad7 (1: 500).

### Data analysis

The software of GraphPad Prism 8.0 (GraphPad Software Inc., California, USA) was used for statistical analysis. Continuous variables were presented as mean ± standard deviation (SD). Statistical differences between groups were evaluated by one-way analysis of variance (ANOVA) followed by least significant difference (LSD) test. The differences were considered statistically significant when *P* value < 0.05.

## Results

### The total polyphenolic glycoside of ***L. rotata*** identified by UPLC- TOF-MS^n^ spectra

**Fig. 1:**
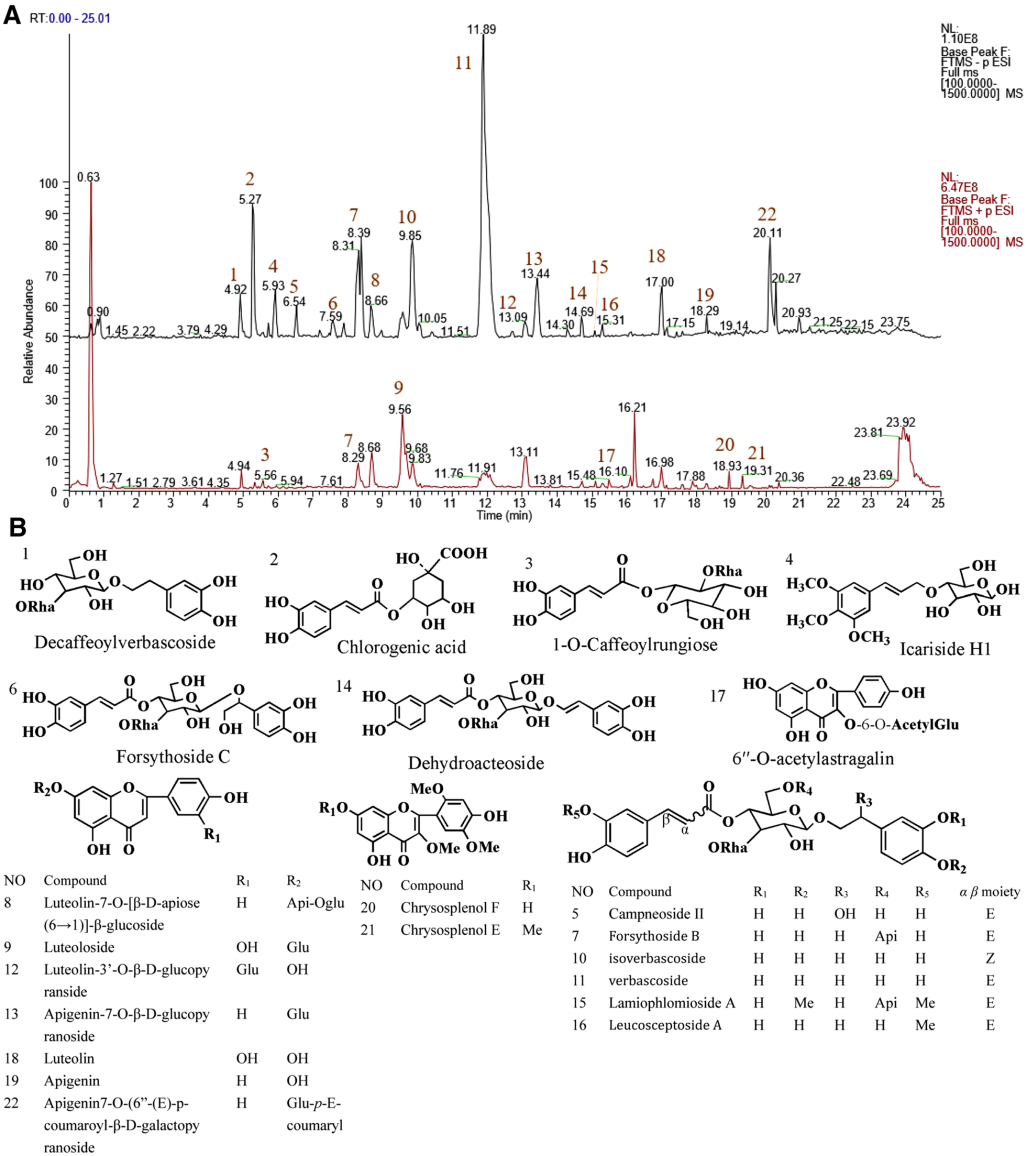
The identification compounds in profile of total phenylpropanoid extract of *L. rotata* (TPLR) by UPLC-Q exactive mass spectrometer. **A** The total ion current in negative and positive mode of TPLR. **B** the chemical structures of compounds identified in TPLR

The total polyphenolic glycoside extract of *L. rotata* (TPLR) was analyzed by MS and MS^n^ in negative and positive ion mode. In total, 24 peaks were detected in the total ion current (TIC) profile of *L. rotata* (Fig. 1A). To identify the compounds, PubChem and SciFinder from the Scholar of the American Chemical Society were used to search for the spectral data of polyphenolic glycosides reported previously in the *L. rotata* and *Lamium* species, and the results were summarized in a spreadsheet (Excel, Microsoft, WA, USA). 22 compounds, including 10 phenylpropanoids and 9 flavonoids, were identified by comparing the retention times and mass spectra of the compounds to those of authentic standards (shown in Fig. 1B and Table 1).

**Table 1:** The identification compounds in total polyphenolic extract of *L. rotata* (TPLR) by UPLC-Q exactive mass spectrometer

Peak Number	RT (min)	Compound	Formula	Calculated (Da)	[M–H]^**−**^	[2 M–H]^**−**^	[M + Na] +	[2 M + Na] +
1	4.92	Decaffeoylverbascoside	C_20_H_30_O_12_	462.17373	461.16620	923.33917	485.16238	947.33582
2	5.27	Chlorogenic acid	C_16_H_18_O_9_	354.09509	353.08731	707.18236	377.08377	731.17810
3	5.56	1-O-Caffeoylrungiose	C_21_H_28_O_13_	488.15300	–	–	511.14163	999.29266
4	5.93	Icariside H1	C_18_H_26_O_9_	386.15769	385.11379	771.30755	–	–
5	6.54	Campneoside II	C_29_H_36_O_16_	640.20034	639.19263	1249.39284	–	–
6	7.56	Forsythoside C	C_29_H_36_O_16_	640.20034	639.19263	1249.39276	663.18854	1303.38794
7	8.29	Forsythoside B	C_34_H_44_O_19_	756.24769	755.23987	–	779.23553	–
8	8.68	Luteolin-7-O-[β-D-apiose(6 → 1)]-β-glucoside	C_26_H_28_O_15_	580.14283	579.13525	1159.27661	603.13123	1183.27307
9	9.56	Luteoloside	C_21_H_20_O_11_	448.10057	447.09311	895.19373	471.08926	895.19373
10	9.85	isoverbascoside	C_29_H_36_O_15_	624.20543	623.19788	1247.40161	647.19403	1247.40161
11	11.89	Verbascoside	C_29_H_36_O_15_	624.20543	623.19806	1247.40161	647.19366	1271.39807
12	13.09	Luteolin-3’-O-β-D-glucopyranside	C_21_H_20_O_11_	448.10057	447.09309	895.19376	471.08924	895.19375
13	13.44	Apigenin-7-O-β-D-glucopyranoside	C_21_H_20_O_10_	432.10565	431.09805	863.20319	455.09390	887.19891
14	14.69	Dehydroacteoside	C_29_H_34_O_15_	622.18978	621.18213	1243.37048	645.17816	1267.36707
15	15.08	Lamiophlomioside A	C_36_H_48_O_15_	784.27899	783.27118	–	807.26715	–
16	15.31	Leucosceptoside A	C_30_H_38_O_15_	638.22108	637.21350	1275.43408	661.20966	1299.43030
17	16.12	6′′-O-acetylastragalin	C_22_H_22_O_13_	490.11113	489.10358	979.21387	513.09967	1003.20941
18	17.00	Luteolin	C_15_H_10_O_6_	286.04774	285.04022	571.08765	–	–
19	18.29	Apigenin	C_15_H_10_O_5_	270.05283	269.04553	539.09816	–	–
20	18.93	Chrysosplenol F	C_18_H_16_O_8_	360.08452	359.07700	719.16132	383.07300	743.15747
21	19.31	Chrysosplenol E	C_19_H_18_O_8_	374.10017	–	–	397.08853	771.18829
22	20.11	Apigenin7-O-(6″-(E)-p-coumaroyl-β-D-galactopyranoside	C_30_H_26_O_12_	578.14243	577.35046	1155.27698	601.27307	1179.27234

### TPLR ameliorated hepatic injury and fibrosis

**Fig. 2:**
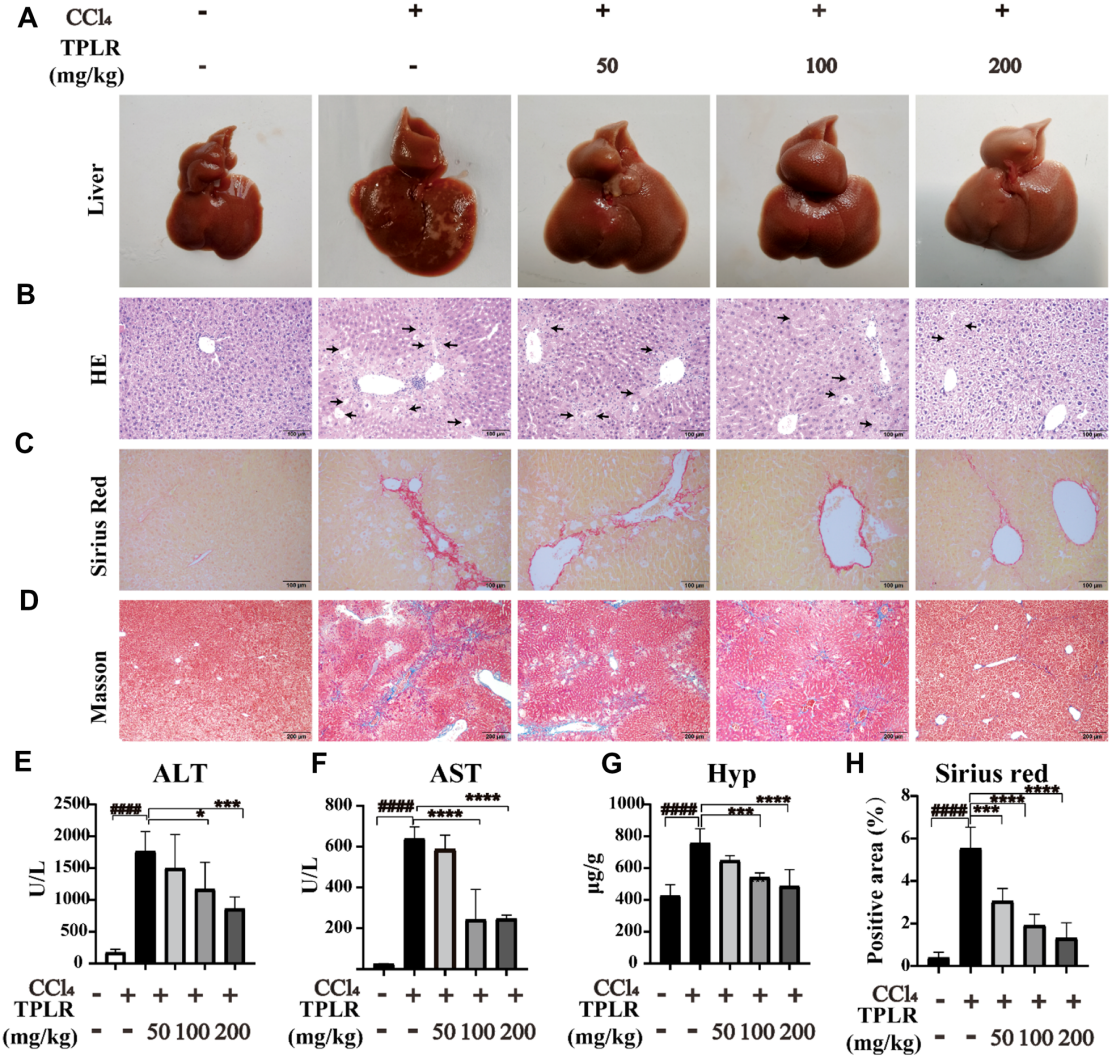
TPLR ameliorated liver injury and reduced hepatic fibrosis in CCl_4_-induced mice. Representative morphology (**A**), HE (**B**), Sirius red (**C**) and Masson trichrome(**D**) staining of liver treatment with or without TPLR, the arrow points to the necrotic site, scale bars of HE, Sirius red staining: 100 μm; scale bars of Masson trichrome staining: 200 μm. The levels of ALT (**E**), AST (**F**) and Hyp (**G**) in serum with or without TPLR treatment. **H** The proportion area statistics of Sirius red. Data were shown as the mean ± SD (n = 6). ^#^*P* < 0.05, vs. control group; ^*^P < 0.05, ^**^P < 0.01, vs. model group

The effects of TPLR against hepatic injury and fibrosis were conducted with CCl_4_ induced hepatic fibrosis which described as reference [34]. The morphology examination of the liver tissue showed that model group with obvious nodular, irregular liver surface, and a wide range of small granular processes. In contrast to model group, TPLR treatment significantly ameliorated the state of fibrosis with tissue swelling (Fig. 2A). Microscopically, HE staining revealed that CCl_4_-induced mice had many fat tissue vacuoles, ballooning of hepatocytes and infiltration of inflammatory cells in the liver tissue, while TPLR exhibited strong efficacy against impaired hepatic tissue (Fig. 2B). Sirius Red and Masson Staining revealed that CCl_4_ injection resulted in connective tissue proliferation and fibrous collagen deposition in the mice liver; in contrast, TPLR reduced the fibrous area in a dose-dependent manner (Fig. 2C, D). Specially, the positive area of Sirius red staining was significantly decreased in the TPLR treatment groups, which suggested that TPLR down-regulated the expression of Collagen I (Col1α1) in liver (Fig. 2H) [35]. Furthermore, the result also showed that TPLR (100, 200 mg/kg) dramatically reduced the levels of ALT (Fig. 2E), AST (Fig. 2F), and Hyp (Fig. 2G). Altogether, these results clearly indicate the hepatoprotection and anti-hepatic fibrosis effects of TPLR.

### TPLR inhibited HSC cells viability and ECM secretion

**Fig. 3:**
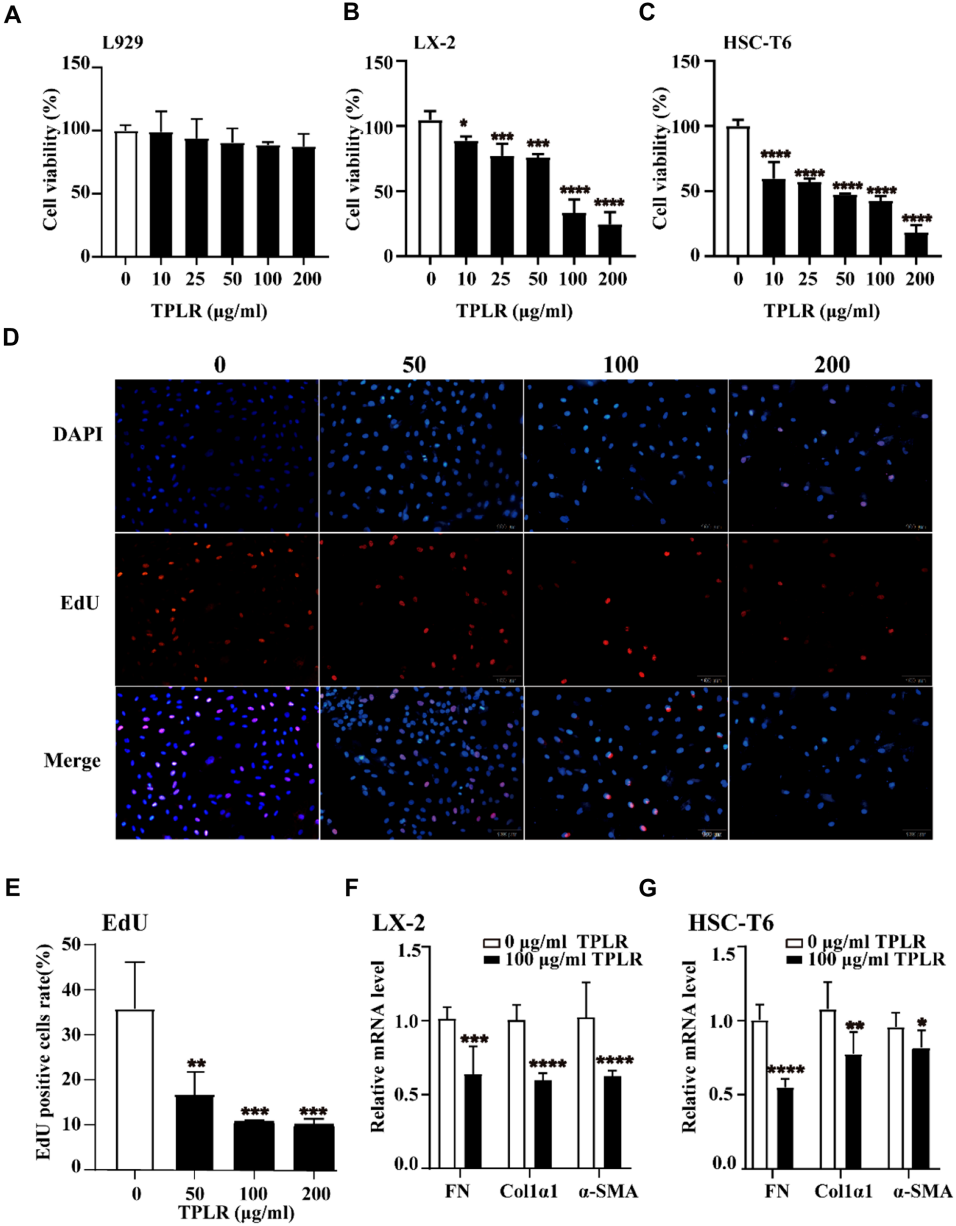
TPLR inhibited the proliferation and secretion on HSCs. The CCK-8 assay of the proliferation on **A** L929, **B** LX-2 and **C** HSC-T6 cells with different concentrations of TPLR (0, 10, 25, 50, 100 and 200 μg/ml) incubated for 48 h (n = 4). **D** The images of EdU staining on LX-2 cells with TPLR (0, 50, 100 and 200 μg/ml) for 48 h. In the representative EdU images, total cells stained with DAPI were blue, whereas proliferating cells were shown as red point, and the ratio of red in blue cells (n = 3), scale bars: 100 μm. **E** Quantitative analysis of EdU staining of proliferation on the LX-2 cells with different concentrations of TPLR treatment (n = 3) (**F**–**G**) The mRNA level of FN, Col1α1 and α-SMA in LX-2 cells with 100 μg/ml TPLR for 48 h. Data were shown as mean ± SD. ^*^P < 0.05, ^**^P < 0.01, ^***^P < 0.001 ^****^P < 0.0001, vs. control group

The cytotoxic effects of TPLR on L929 cells were examined by CCK-8 assay [30]. The CCK-8 assay showed that in these concentrations, no significant cytotoxic effect of TPLR was obvious on L929 cells for 48 h (Fig. 3A). Since HSCs are critical for hepatic fibrogenesis, along with an increase in cell proliferation and oversecretion of extracellular matrix [36], two lines of HSC (LX-2 and HSC-T6 cells) was employed to measure the viability of TPLR on HSCs, TPLR significantly inhibited cell proliferation on LX-2 and HSC-T6 cells in a concentration-dependent manner (Fig. 3B, C), and the IC_50_ values were 140.8 and 78.12 μg/mL, respectively. Human cells, as opposed to rodent cells, are preferred for clarifying the mechanism of the antifibrotic drug, which could continuously maintain the in vivo phenotype [37, 38], we further investigated the effects of TPLR on proliferation in LX-2 by EdU staining, consistent with the results of CCK-8 assay, TPLR also showed the repression of proliferation on LX-2 cells with EdU staining (Fig. 3D, E). Moreover, in order to clarify the effect of TPLR on the ECM secretion of HSCs, we examined amplification of FN, COL1α1 and α-SMA in LX-2 and HSC-T6 cells by qPCR. Indeed, the mRNA levels of FN, COL1α1 and α-SMA were significantly down-regulated in LX-2 and HSC-T6 cells treated with 100 μg/mL TFLR for 48 h (Fig. 3F, G). These results indicated that TPLR inhibited the cells proliferation and ECM deposition both in LX-2 and HSC-T6 cells.

### TPLR promoted the apoptosis of LX-2 cells

**Fig. 4:**
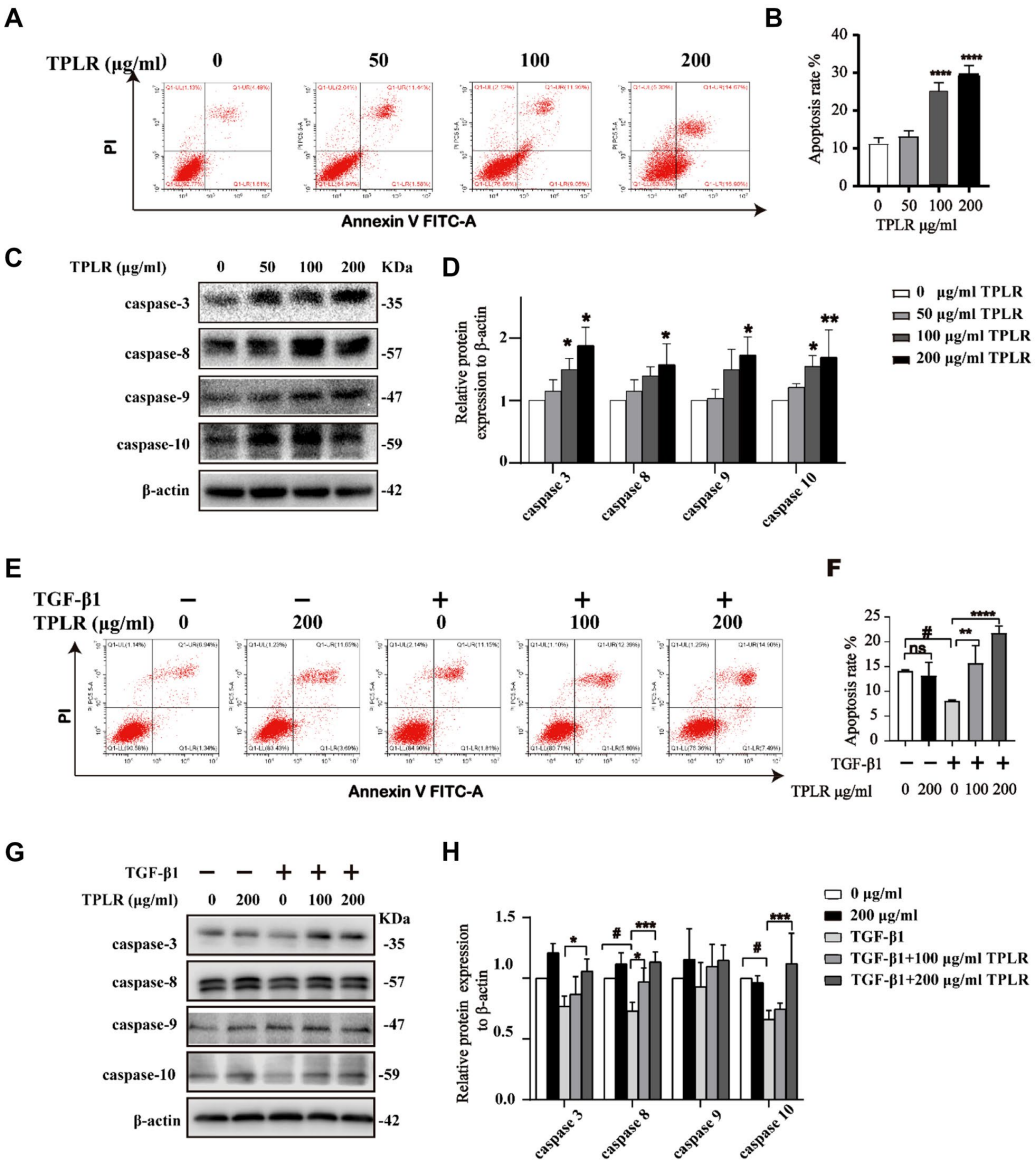
TPLR significantly induced the apoptosis on LX-2 cells. **A** The apoptosis effect of LX-2 cells with 0, 50, 100 or 200 μg/ml TPLR for 48 h by flow cytometry. **B** Quantitative analysis of **A**. **C** The levels of caspase-3, -8, -9, -10 after an incubation with various concentrations of TPLR (0, 50, 100 or 200 μg/ml) for 48 h by Western blot. **D** Quantitative analysis for western blotting (n = 3). The results are shown as mean ± SD; *P < 0.05, **P < 0.01, compared with the control group. **E** The apoptosis rates of LX-2 cells with TPLR (0, 100 or 200 μg/ml) for 12 h in the presence of TGF-β1 (10 ng/ml) for 12 h by flow cytometry. **F** Quantitative analysis of **E**. **G** The western blot results of the caspase family in LX-2 cells with TPLR (0, 50, 100 or 200 μg/ml) for 12 h with 10 ng/ml TGF-β1. **H** Quantitative analysis for western blotting (n = 3). The results are shown as mean ± SD; ^#^*P* < 0.05, compared with the control group; *P < 0.05, **P < 0.01, ***P < 0.001 compared with the TGF-β1 group

To explore the possible mechanism of TPLR inhibiting the proliferation of LX-2 cells, we investigated the cell cycle and apoptosis the effect of HSCs with TPLR by flow cytometry. The flow cytometry results showed that TPLR had no obvious effect on the cell cycle (Additional file 1: Fig. S1), while TPLR significantly increased apoptotic rates of LX-2 cells in a dose-dependent manner at 48 h (Fig. 4A, B). Consequently, we verified the mechanisms of TPLR regulation the apoptosis in LX-2 cells by western blot, the results showed that concentration of TPLR (200 μg/mL) notably enhanced the expression of caspase-3, -8, -9 and -10 after treatment for 48 h (Fig. 4C, D). Since TGF-β1 also plays a vital contributory role in cellular functions such as cell growth, cell differentiation, and apoptosis [39]. We further investigated whether TPLR promoted the apoptosis of activated HSC with TGF-β1 by flow cytometry, the result showed that converse to TGF-β1 resisted apoptosis of LX-2 cells, TPLR significantly elevated the apoptosis rate of activated HSCs in 12 h (Fig. 4E, F). Correspondingly, apoptosis regulatory proteins were detected by western blot assay, as expected, TPLR treatment significantly increased the expression of caspase-3, -8, and -10 at 12 h, which belong to the extrinsic apoptosis subfamily; unexpectedly, TPLR barely raised the expression of caspase-9 (Fig. 4G, H) which the essential initiator caspase required for apoptosis signaling through the mitochondrial pathway [40]. In summary, the result suggested the effects of TPLR promoting the cells apoptosis was regulated by TGF-β1 which are resistant to intrinsic apoptosis pathway.

### TPLR inhibited TGF-β/Smad signaling 

#### *TPLR down-regulated TGF-β/Smad signaling pathway *in vitro

**Fig. 5:**
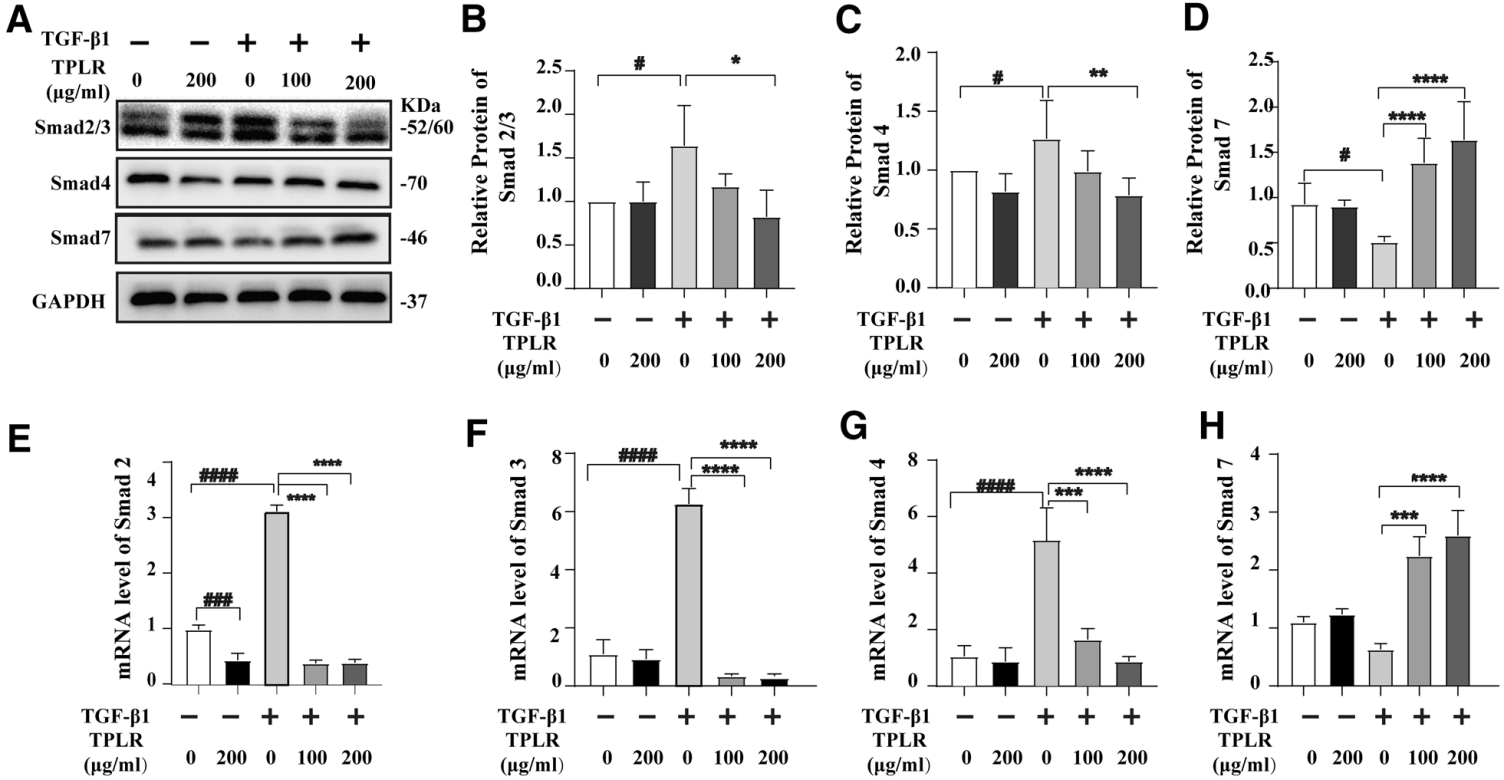
TPLR inhibited the TGF-β/Smad signaling pathway on LX-2 cells. **A** The protein levels of Smad2/3, Smad4, and Smad7 with TPLR (0, 100 and 200 μg/ml) for 12 h by Western blot. **B**–**D** Quantitative analysis for western blotting of **B** Smad2/3, **C** Smad4 and **D** Smad7 (n = 3). The mRNA levels of **E** Smad4, **F** Smad2, **G** Smad3, and **H** Smad7 with or without TPLR by qPCR (n = 3). Data were shown as mean ± SD. ^#^*P* < 0.05, vs. control group; ^*^P < 0.05, ^**^P < 0.01, ^****^P < 0.0001 vs. TGF-β1 group

TGF-β1 also has been characterized as a critical mediator in the process of HSC activation [41], we further explore the mechanism of TPLR inhibited HSCs activation by target TGF-β/Smad signaling. Serum-starved LX-2 cells were treated with 10 ng/mL TGF-β1 for 12 h followed by TPLR for 12 h, the expression of Smad2/3 and Smad4 was increased by TGF-β1 treatment, and TPLR treatment significantly reversed this change (Fig. 5A–C). Meanwhile, the expression of Smad7, which is an antagonist of TGF-β/Smad signaling, was decreased with TGF-β1 stimulation in LX-2 cells, and TPLR treatment significantly increased the protein expression of Smad7 (Fig. 5A, D). Fortunately, similar changes were also observed at the mRNA levels of Smad2/3, Smad4 and Smad7 by qPCR (Fig. 5E–H). Altogether, these results indicated that TPLR notably restrained TGF-β/Smad signaling in vitro.

#### *TPLR down-regulated TGF-β/Smad signaling pathway *in vivo

**Fig. 6:**
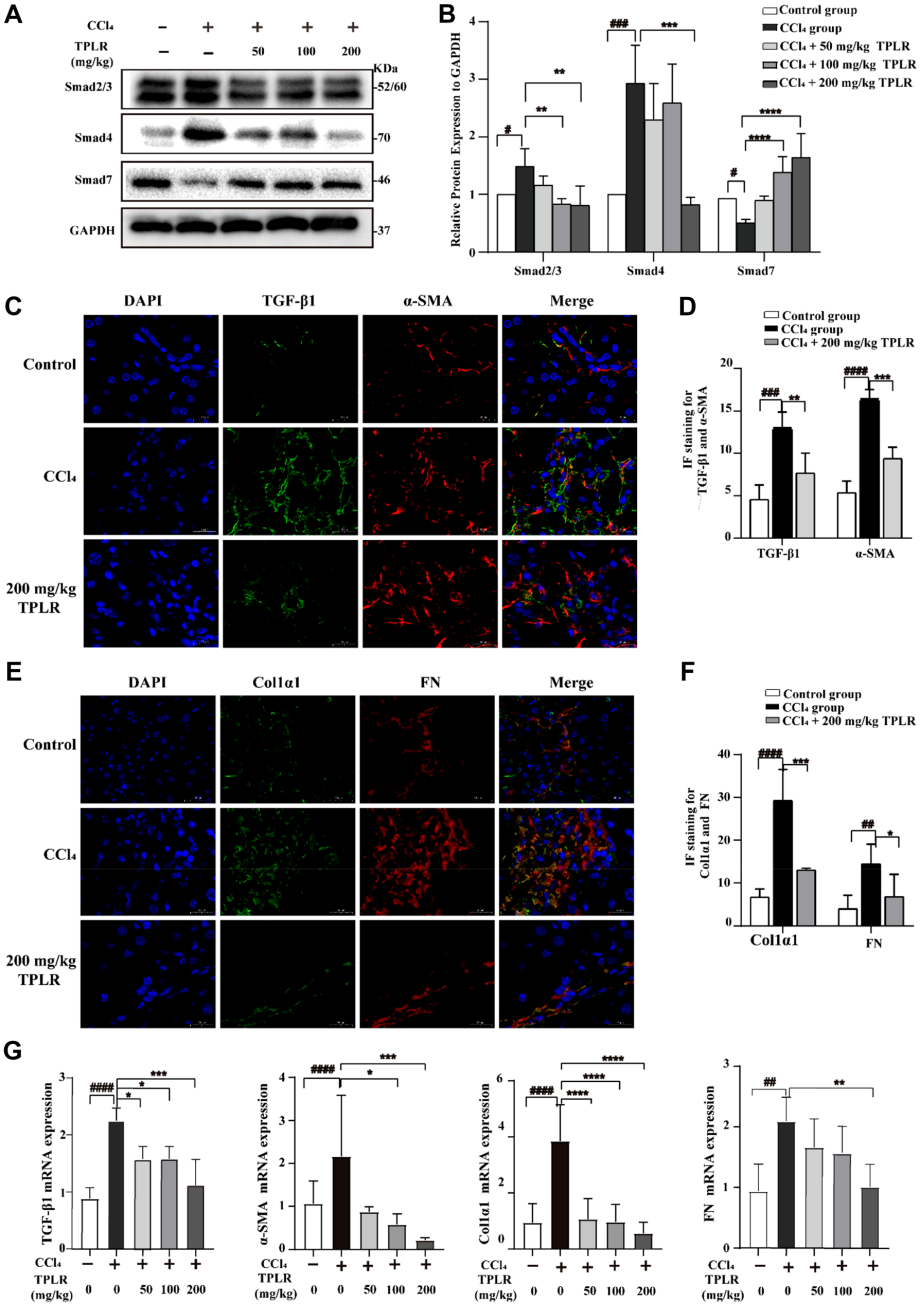
TPLR downregulated TGF-β/Smad signaling pathway in mice. **A** The expressions of Smad4, Smad2/3 and Smad7 in liver with or without TPLR treatment by western blotting. **B** Quantitative analysis for western blotting of Smad2/3, Smad4 and Smad7 (n = 3). **C** Dual immunofluorescence staining for TGF-β1 (green) and α-SMA (red) in mice liver tissues with or without TPLR treatment. scale bar, 20 μm. **D** Quantitative analysis of **C**. **E** Dual immunofluorescence staining for Col1α1(green) and FN (red) in mice liver tissues with or without TPLR treatment. scale bar, 20 μm. **F** Quantitative analysis of **E**. Images are representative of 3 mice per group. **G** The mRNA levels of TGF-β1, α-SMA, Col1α1 and FN with or without TPLR treatment by qPCR (n = 6). ^*###*^*P* < 0.001, ^####^*P* < 0.0001 vs. normal group; ^*^P < 0.05, ^**^P < 0.01, vs. model group

The progression and regression of hepatic fibrosis rely on a complex interplay of the hepatic microenvironment [42], to further explore whether TPLR inhibit the TGF-β/Smad signaling pathway under the internal environment, we examined the antifibrotic effect of TPLR on the CCl_4_-induced fibrotic mice, eight weeks after the repetitive injection of CCl_4_, proteins of Smad2/3 and Smad4 were significantly expressed, and Smad7 was obviously decreased in the model group. Compared to hepatic fibrotic mice, TPLR significantly down-regulated the protein expression levels of Smad2/3 and Smad4 and up-regulated Smad7 in a dose-dependent manner (Fig. 6A, )B). A hallmark of fibrosis is the overexpression of α-SMA and deposition of ECM, which mainly consists of Col1α1 and FN [43]. In this study, IF stains showed high levels of TGF-β1, α-SMA, COL1α1 and FN in the model group, whereas the deposition of extracellular matrix (ECM) was remarkably decreased in TPLR group (Fig. 6C-F). Further quantification of the mRNA levels in the tissue indicated that TGF-β1, α-SMA, COL1α1 and FN expression were significantly decreased compared to the control group (Fig. 6G). Based on these data, TPLR markedly decreased ECM deposition, ameliorated hepatic fibrogenesis by downregulating TGF-β/Smad signaling pathway in the hepatic tissue.

## Discussion

Liver fibrosis is caused by various chronic liver diseases, and eventually progress to cirrhosis and even HCC, and the activated HSCs plays a key role in the development of liver fibrosis [44]. Clinical and experimental studies have demonstrated that removal of the etiologic stimuli of liver injury results in regression of liver fibrosis and disappearance of activated HSCs by apoptosis [45], and it be beneficial to investigate compounds or extracts which promote the apoptosis of activated HSCs. Fortunately, TPLR treatment was able to significantly inhibited HSC activation in vitro, as evidenced by its inhibitory effect on the expression of the activation marker a-SMA, collagen as well as other fibrotic genes. Moreover, Caspases (cysteine-aspartic proteases) are proteolytic enzymes largely known for their role in controlling cell death, once initiator caspases are activated through the extrinsic or intrinsic apoptosis pathways, they mediate activation of effector caspases-3 [46]. In this study, TPLR (200 μg/mL) significantly increased the expression of caspase-10, -8 and -3 at 12 h after HSCs activation with TGF-β1, the result suggested TPLR promoted apoptosis through an extrinsic apoptotic pathway under TGF-β1 stimulation [47]. However, these apoptosis proteins were scarcely up-regulated with TPLR direct intervention for 12 h; surprisingly, after TPLR treatment 48 h, the expression of caspase-3, -8, -10 and -9 were significantly increased, since caspase-9 plays a central role of intrinsic apoptotic pathway [48, 49], the result suggested the apoptosis of HSCs were both in intrinsic and extrinsic apoptotic pathway with TPLR direct intervention. Altogether, our results suggested that TPLR promoted apoptosis of HSCs.

TGF-β1 also activates the downstream signaling pathway, promotes the phosphorylation of Smad2/3, and binds to Smad4 in the nucleus to mediate fibrosis, which is negatively regulated by the Smad7 degradation pathway [41]. Our research proved that TPLR inhibited TGF-β1 signaling pathway by downregulating the Smad2/3 and Smad4 in a dose-dependent manner. Importantly, Smad7 inhibits TGF-β1 signaling pathway and mediates apoptosis in various cells, recent studies have confirmed that upregulation Smad7 promoted the apoptosis of podocytes [50], ovarian granulosa cells [51] and HSCs [52]. Consistent with these studies, we also found that TPLR significantly increased the expression of Smad7 in vivo and in vitro, it suggested that TPLR ameliorates hepatic fibrosis and promoted the apoptosis of HSCs mainly depended on the TGF-β/Smad signaling pathway.

To investigate the effective chemicals in TPLR extract, the chemical constituents of TPLR were identified with an UPLC-TOF-MS^n^ spectra method. In this study, verbascoside was found to be the main compounds of TPLR extract since the area of the compound accounts for 25.6% in the total ion current profile, meanwhile, several studies revealed that verbascoside promoted apoptosis in a variety of tumor cells, such as such as oral squamous cells [53], human colorectal cancer [54], and glioblastoma [55], these results inferred that verbascoside would be one of the main bioactive compounds which was responsible for promoting apoptosis effect of TPLR. Interestingly, compared with the model group, we also found TPLR (200 mg/ml) down-regulated the expression of apoptosis regulatory proteins in liver which suggested that TPLR could protect mice from CCl_4_-induced liver steatosis (Additional file 1: Fig. S2) [56]. Furthermore, among the identified compounds in TPLR, luteolin was listed as the marker compound used for quality control during 1995–2000 in the Chinese pharmacopoeia [19], and many studies demonstrated that the compound exhibited antifibrotic effects by downregulation TGF-β/Smad signaling pathway in recent years [57, 58]. From the above mentioned, our study confirmed TPLR induced the apoptosis of HSCs via TGF-β/Smad signaling pathway against hepatic fibrosis.

## Conclusion

In vitro and in vivo studies have shown that TPLR, a total polyphenolic glycoside extracted of* L rotata*, is the main active substance against hepatic fibrosis, which promotes the apoptosis of HSCs by TGF-β/Smad signaling pathway.

## Supplementary Information

**Additional file 1:** **Table S1.** List of primers in PCR amplification. **Fig. S1.** The effect of TPLR on the cell cycle of LX-2 cells. **Fig. S2.** TPLR downregulated apoptosis-related proteins in mice. **Fig. S4.** Original images of Western Blot.

## Data Availability

The raw data for this article will be made available by the authors, without undue reservation.

## Abbreviations

ECM: extracellular matrix
HSCs: Hepatic stellate cells
*L. rotata*: *Lamiophlomis rotate*
TPLR: Total polyphenolic glycosides of *L. rotate*
UPLC-Q/TOF/MS^n^: Ultrahigh-performance liquid chromatography coupled with time-of-flight mass spectrometry
CCl_4_: Carbon tetrachloride
FN: Fibronectin
α-SMA: α-Smooth muscle actin
Col1α1: Collagen I
HCC: Hepatocellular carcinoma
TRAIL: TNF-related apoptosis-inducing ligand
TGF-β: Transforming growth factor-β
AST: Serum aspartate aminotransferase
ALT: Alanine aminotransferase
Hyp: Hydroxyproline
DMEM: Dulbecco’s modified Eagle’s medium
PMSF: Phenylmethanesulfonyl fluoride
PVDF: Polyvinylidene fluoride membranes
CMC-Na: Sodium carboxymethylcellulose
HE: Hematoxylin and eosin
IF: Immunofluorescence
LSD: Least significant difference
