# Supplementary material for: The total polyphenolic glycoside extract of Lamiophlomis rotata ameliorates hepatic fibrosis through apoptosis by TGF-β/Smad signaling pathway
Source: Chin Med. 2023 Feb 24;18:20. doi: 10.1186/s13020-023-00723-x (PMC9951520; doi:10.1186/s13020-023-00723-x)
Supplement: Supplementary file 1 — Additional file 1: Table S1. List of primers in PCR amplification. Fig. S1. The effect of TPLR on the cell cycle of LX-2 cells. Fig. S2. TPLR downregulated apoptosis-related proteins in mice. Fig. S4. Original images of Western Blot. [file 13020_2023_723_MOESM1_ESM.docx]

Additional Material

# Additional Figures and Tables

## Additional Figures


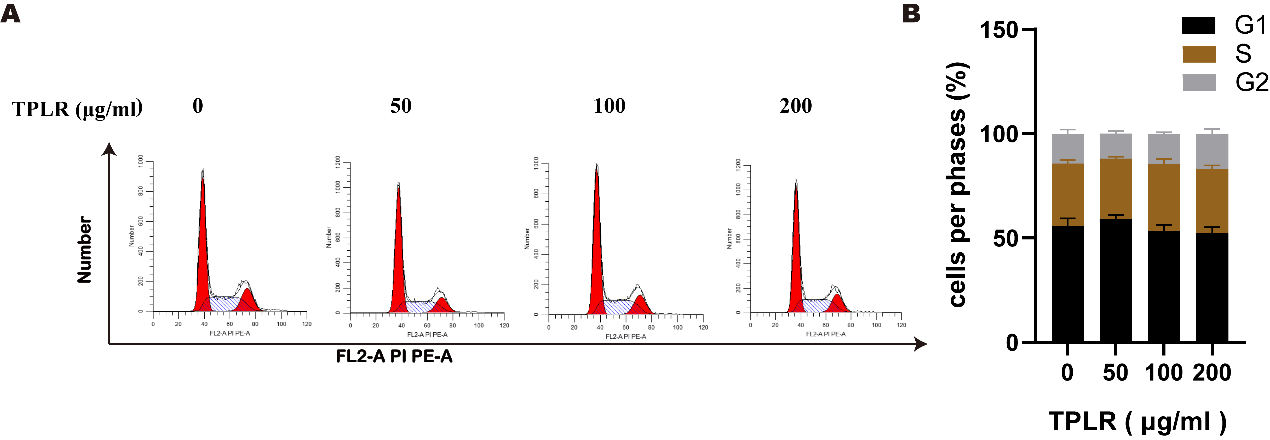


## Supplementary Fig. 1 TPLR had no obvious effect of TPLR on the cell cycle of LX-2 cells. (A) LX-2 cells were treated with 0, 50, 100 or 200 μg/ml TPLR for 48 h, cell per phases was measured by flow cytometry. (B) Quantitative analysis of the cell per phases of LX-2 cells induced by TPLR with flow cytometry assay.

## 1.2 Additional Figures


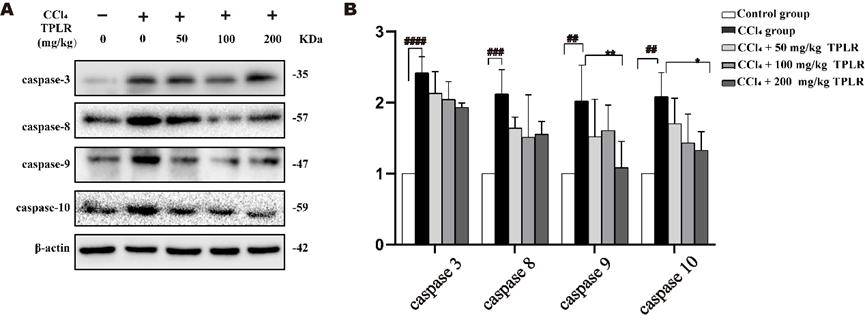


Supplementary Fig. 4 TPLR downregulated apoptosis-related proteins in mice. (A) The expressions of caspase-3, -8, -9 and -10 in liver with or without TPLR treatment by western blotting. (B) Quantitative analysis for western blotting of caspase-3, -8, -9 and -10 (n=3). *^###^P* < 0.001, ^####^*P* < 0.0001 vs. normal group; ^*^*P* < 0.05, ^**^*P* < 0.01, vs. model group.

## 1.3 Additional Tables

## Supplementary Table 1. List of primers in PCR amplification

| Gene name | Resource | Forward | Reverse |
| --- | --- | --- | --- |
| GAPDH | Homo | TCTGACTTCAACAGCGACACC | CTGTTGCTGTAGCCAAATTCGTT |
| Col 1α1 | Homo | GTTTGCTAATGCTGCTCCCG | CATGACCGAGACGTGTGGAA |
| FN | Homo | ACTCACCAGTGTGGTCTGTG | GGACGTTCTACTCCTGCACC |
| α-SMA | Homo | CAGGGCTGTTTTCCCATCCAT | GCCATGTTCTATCGGGTACTTC |
| SMAD2 | Homo | AGGGGCAGAAAACAACTCT | ATGCGCTCCACTACTGAAA |
| SMAD3 | Homo | TGAATGTTGGTGGAGGGT | CGGCCACTTGTTTAGCC |
| SMAD4 | Homo | GCTGCTGTCCTATGCTTTG | GCGGTCCTCCAAGTGAT |
| SMAD7 | Homo | AGAAGGTGCGGAGCAAAA | GTGTGGCGGACTTGATGA |
| GAPDH | Rno | TCTGACTTCAACAGCGACACC | CTGTTGCTGTAGCCAAATTCGTT |
| Col 1α1 | Rno | GGAGAGAGCATGACCGATGG | GGGACTTCTTGAGGTTGCCA |
| α-SMA | Rno | CGAAGCGCAGAGCAAGAGA | CATGTCGTCCCAGTTGGTGAT |
| FN | Rno | TGGAGAGACAGGAGGAAATAGC | CAGTGACAGCATACAGGGTGAT |
| GAPDH | Mice | TGTTTCCTCGTCCCGTAGA | ATCTCCACTTTGCCACTGC |
| α-SMA | Mice | GCCCAGAGCAAGAGAGG | TGTCAGCAGTGTCGGATG |
| TGF-β1 | Mice | CCATTGCTGTCCCGTGCAGA | CGAAAGCCCTGTATTCCGTCT |
| Col 1α1 | Mice | CAGAGGCGAAGGCAACA | GTCCAAGGGAGCCACATC |
| FN | Mice | TGCCGTGGTCCTAACAA | GACCTGTTTTCTGCCTTCC |

## 1. 4 Original images of Western Blot in this study

## Original images of Western Blot in Fig. 4C

| 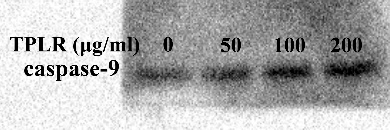 | 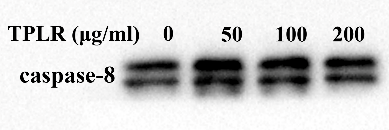 | 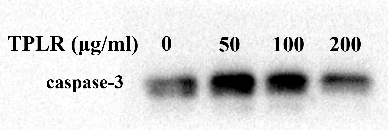 |
| --- | --- | --- |
| 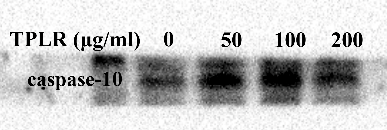 | 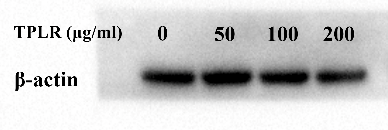 |  |

## Original images of Western Blot in Fig. 4G

| 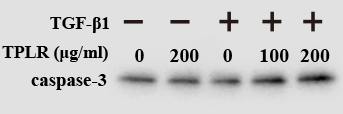 | 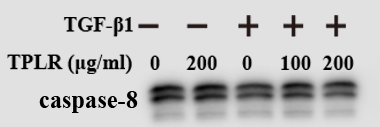 | | 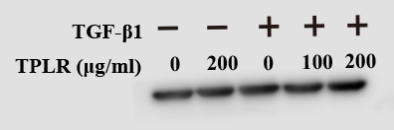 |
| --- | --- | --- | --- |
| 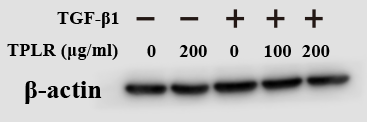 | 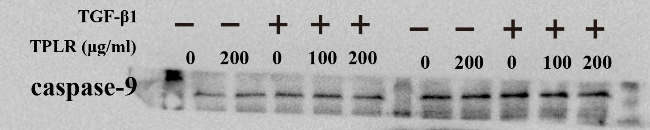 | | |
| 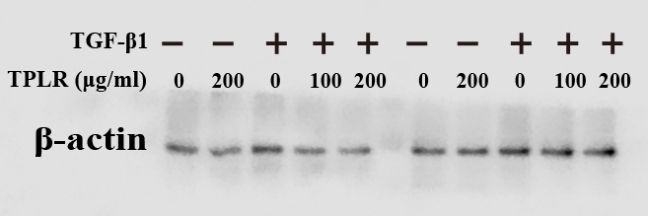 | | 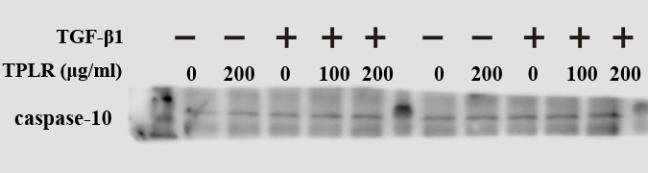 | |

## Original images of Western Blot in Fig. 5A

| 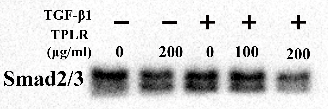 | 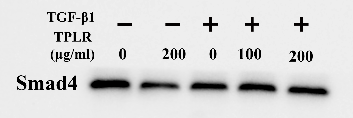 | 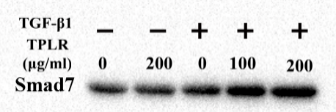 |
| --- | --- | --- |
| 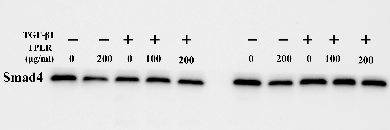 | 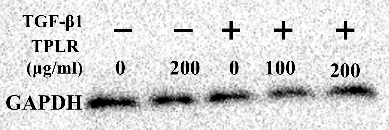 |  |

## Original images of Western Blot in Fig. 6A

| 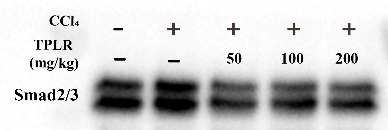 | 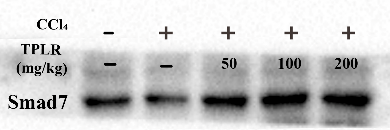 |
| --- | --- |
| 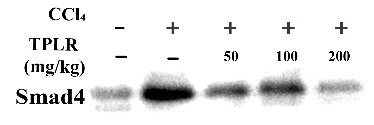 | 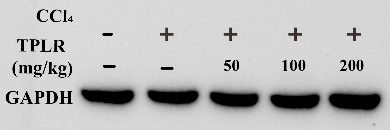 |
